# Supplementary material for: Impact of sequential herbicides application on crop productivity, weed and nutrient dynamics in soybean under conservation agriculture in Vertisols of Central India
Source: PLoS One. 2023 Jan 20;18(1):e0279434. doi: 10.1371/journal.pone.0279434 (PMC9857987; doi:10.1371/journal.pone.0279434)
Supplement: Attachment — Submitted filename: PONE-D-22-19003_reviewer reviewed.pdf [file pone.0279434.s002.pdf]

PLOS ONE

Impact of sequential herbicides application on crop productivity, weed and nutrient dynamics in soybean under conservation agriculture in Vertisols of Central India

--Manuscript Draft--

**Manuscript Number:**	PONE-D-22-19003
**Article Type:**	Research Article
**Full Title:**	Impact of sequential herbicides application on crop productivity, weed and nutrient dynamics in soybean under conservation agriculture in Vertisols of Central India
**Short Title:**	Impact of sequential herbicides on weed and nutrient dynamics in soybean under conservation agriculture
**Corresponding Author:**	Bharat Prakash Meena, Ph.D.<br>Indian Institute of Soil Science<br>bhopal, INDIA
**Keywords:**	Conservation agriculture, soybean, weed management, nutrient dynamics, Vertisols
**Abstract:**	Adoption of conservation agriculture (CA) is very slow due to weed infestations. The application of herbicides is the only viable option to deal with problem of weed management to adhere with basic principles of CA. A field experiment was carried out for three years to evaluate the expediency of different herbicides and their sequential applications under CA. In this study, seven treatments comprised of either alone or sequential application of pre-emergence (PE) and post-emergence (PoE) herbicides, hand weeding and weedy check were tested in soybean. Result indicated that sequential application of glyphosate at 1kg ai ha^{-1} + pendimethalin at 1kg ai ha^{-1} as PE followed by PoE application of imazethapyr at 100 g ai ha^{-1} at 30 days after sowing (DAS) proved to be the best economical option in terms of plant growth parameters, crop biomass, seed yield, weed index and carbon and nutrient recycling. Pearson's correlation coefficients matrix revealed that grain yield was significantly ($P<0.0001$) related to weed density ($r=-0.84$), (WDH) ($r=-0.63$), weed dry biomass (WDB) ($r=-0.52$), weed nitrogen (N), phosphorus (P) and potassium (K) uptake ($r=-0.56$, $r=-0.59$ and $r=-0.66$) and weed index (WI) ($r=-0.96$). The bivariate linear regression study of grain yield on weed control efficiency biomass, N, P and K uptake by grain showed a clear significant ($P<0.0001$) dependence with R^2 value of 0.53, 0.99, 0.95 and 0.98, respectively. The fitted stepwise multiple regression model also revealed that N and P uptake in grain, weed density at 20 DAS and K uptake in weed were actual predictor for grain yield. We concluded that, effective and economical weed control under CA in soybean can be achieved through sequential application of glyphosate along with pendimethalin at 1 kg ai ha^{-1} as PE followed by PoE use of imazethapyr at 100 g ai ha^{-1} at 30 DAS.
**Order of Authors:**	A K. Vishwakarma, Ph.D.
	Bharat Prakash Meena, Ph.D.
	Hiranmoy Das, Ph.D.
	Pramod Jha, Ph.D.
	A. K. Biswas, Ph.D.
	K. Bharati, Ph.D.
	K. M. Hati, Ph.D.
	R. S. Chaudhary, Ph.D.
	A. O. Shirale, Ph.D.
	B. L. Lakaria, Ph.D.
	Priya P Gurav, Ph.D.
	Ashok K Patra, Ph.D.
**Additional Information:**	

Powered by Editorial Manager® and ProduXion Manager® from Aries Systems Corporation

Question	Response
**Financial Disclosure**<br><br>Enter a financial disclosure statement that describes the sources of funding for the work included in this submission. Review the submission guidelines for detailed requirements. View published research articles from *PLOS ONE* for specific examples.<br><br>This statement is required for submission and **will appear in the published article** if the submission is accepted. Please make sure it is accurate.<br><br>**Unfunded studies**<br>Enter: *The author(s) received no specific funding for this work.*<br><br>**Funded studies**<br>Enter a statement with the following details:<br>• Initials of the authors who received each award<br>• Grant numbers awarded to each author<br>• The full name of each funder<br>• URL of each funder website<br>• Did the sponsors or funders play any role in the study design, data collection and analysis, decision to publish, or preparation of the manuscript?<br>• **NO** - Include this sentence at the end of your statement: *The funders had no role in study design, data collection and analysis, decision to publish, or preparation of the manuscript.*<br>• **YES** - Specify the role(s) played.<br><br>* typeset	The author(s) received no specific funding for this work.
**Competing Interests**<br><br>Use the instructions below to enter a competing interest statement for this submission. On behalf of all authors, disclose any competing interests that could be perceived to bias this work—acknowledging all financial support and any other relevant financial or non-financial competing interests.	The authors have declared that no competing interests exist.

This statement is required for submission and **will appear in the published article** if the submission is accepted. Please make sure it is accurate and that any funding sources listed in your Funding Information later in the submission form are also declared in your Financial Disclosure statement.<br><br>View published research articles from *PLOS ONE* for specific examples.<br><br>**NO authors have competing interests**<br><br>Enter: *The authors have declared that no competing interests exist.*<br><br>**Authors with competing interests**<br><br>Enter competing interest details beginning with this statement:<br><br>*I have read the journal's policy and the authors of this manuscript have the following competing interests: [insert competing interests here]*<br><br>* typeset	
**Ethics Statement**<br><br>Enter an ethics statement for this submission. This statement is required if the study involved:<br><br>• Human participants<br>• Human specimens or tissue<br>• Vertebrate animals or cephalopods<br>• Vertebrate embryos or tissues<br>• Field research<br><br>Write "N/A" if the submission does not require an ethics statement.<br><br>General guidance is provided below. Consult the submission guidelines for detailed instructions. **Make sure that all information entered here is included in the Methods section of the manuscript.**	N/A

**Format for specific study types**<br><br>**Human Subject Research (involving human participants and/or tissue)**<br>• Give the name of the institutional review board or ethics committee that approved the study<br>• Include the approval number and/or a statement indicating approval of this research<br>• Indicate the form of consent obtained (written/oral) or the reason that consent was not obtained (e.g. the data were analyzed anonymously)<br><br>**Animal Research (involving vertebrate animals, embryos or tissues)**<br>• Provide the name of the Institutional Animal Care and Use Committee (IACUC) or other relevant ethics board that reviewed the study protocol, and indicate whether they approved this research or granted a formal waiver of ethical approval<br>• Include an approval number if one was obtained<br>• If the study involved *non-human primates*, add *additional details* about animal welfare and steps taken to ameliorate suffering<br>• If anesthesia, euthanasia, or any kind of animal sacrifice is part of the study, include briefly which substances and/or methods were applied<br><br>**Field Research**<br><br>Include the following details if this study involves the collection of plant, animal, or other materials from a natural setting:<br>• Field permit number<br>• Name of the institution or relevant body that granted permission	
**Data Availability**<br><br>Authors are required to make all data underlying the findings described fully available, without restriction, and from the time of publication. PLOS allows rare exceptions to address legal and ethical concerns. See the PLOS Data Policy and FAQ for detailed information.	Yes - all data are fully available without restriction

Powered by Editorial Manager® and ProduXion Manager® from Aries Systems Corporation

A Data Availability Statement describing where the data can be found is required at submission. Your answers to this question constitute the Data Availability Statement and **will be published in the article**, if accepted.<br><br>**Important:** Stating 'data available on request from the author' is not sufficient. If your data are only available upon request, select 'No' for the first question and explain your exceptional situation in the text box.<br><br>Do the authors confirm that all data underlying the findings described in their manuscript are fully available without restriction?	
**Describe where the data may be found in full sentences. If you are copying our sample text, replace any instances of XXX with the appropriate details.**<br><br>• If the data are **held or will be held in a public repository**, include URLs, accession numbers or DOIs. If this information will only be available after acceptance, indicate this by ticking the box below. For example: *All XXX files are available from the XXX database (accession number(s) XXX, XXX.).*<br>• If the data are all contained **within the manuscript and/or Supporting Information files**, enter the following: *All relevant data are within the manuscript and its Supporting Information files.*<br>• If neither of these applies but you are able to provide **details of access elsewhere**, with or without limitations, please do so. For example:<br><br>*Data cannot be shared publicly because of [XXX]. Data are available from the XXX Institutional Data Access / Ethics Committee (contact via XXX) for researchers who meet the criteria for access to confidential data.*<br><br>*The data underlying the results presented in the study are available*	All relevant data are within the manuscript and its Supporting Information files.

from (include the name of the third party and contact information or URL).<br>• This text is appropriate if the data are owned by a third party and authors do not have permission to share the data.<br><br>* typeset	
Additional data availability information:	

Manuscript

Click here to access/download;Manuscript;Manuscript.docx

Impact of sequential herbicides application on crop productivity, weed and nutrient dynamics in soybean under conservation agriculture in Vertisols of Central India

A.K. Vishwakarma[1], Bharat Prakash Meena[1]*, Hiranmoy Das[2], Pramod Jha[1], A.K. Biswas[1], K. Bharati[3], K.M. Hati[4], R.S. Chaudhary[4], AO Shirale[1], B.L. Lakaria[1], Priya P. Gurav[1], Ashok K. Patra[5]

[1]Divison of Soil Chemistry & Fertility, ICAR–Indian Institute of Soil Science, Bhopal, 462 038, India

[2] All India Coordinated Research Project on Soil Test Crop Response, ICAR–Indian Institute of Soil Science, Bhopal, 462 038, India

[3]Division of Soil Biology, ICAR–Indian Institute of Soil Science, Bhopal, 462 038, India

[4]Division of Soil physics, ICAR–Indian Institute of Soil Science, Bhopal, 462 038, India

[5]Director, ICAR–Indian Institute of Soil Science, Bhopal, 462 038, India

ABSTRACT

Adoption of conservation agriculture (CA) is very slow due to weed infestations. The application of herbicides is the only viable option to deal with problem of weed management to adhere with basic principles of CA. A field experiment was carried out for three years to evaluate the expediency of different herbicides and their sequential applications under CA. In this study, seven treatments comprised of either alone or sequential application of pre-emergence (PE) and post-emergence (PoE) herbicides, hand weeding and weedy check were tested in soybean. Result indicated that sequential application of glyphosate at 1kg ai ha^{-1} + pendimethalin at 1kg ai ha^{-1}as PE followed by PoE application of imazethapyr at 100 g ai ha^{-1} at 30 days after sowing (DAS) proved to be the best economical option in terms of plant growth parameters, crop biomass, seed yield, weed index and carbon and nutrient recycling. Pearson's correlation coefficients matrix revealed that grain yield was significantly (P<0.0001) related to weed density (r=-0.84), (WDI) (r=-0.63), weed dry biomass (WDB) (r=-0.52), weed nitrogen (N), phosphorus (P) and potassium (K) uptake (r=-0.56, r=-0.59 and r=-0.60) and weed index (WI) (r=-0.96). The bivariate linear regression study of grain yield on weed control efficiency (WCI) biomass, N, P and K uptake by grain showed a clear significant (P<0.0001) dependence with R^2 value of 0.53, 0.99, 0.95 and 0.98, respectively. The fitted stepwise multiple regression model also revealed that N and P uptake in grain, weed density at 20 DAS and K uptake in weed were actual predictor for grain yield. We concluded that, effective and economical weed control under CA in soybean

can be achieved through sequential application of glyphosate along with pendimethalin at 1 kg ai ha^{-1} as PE followed by PoE use of imazethapyr at 100 g ai ha^{-1} at 30 DAS.

KEYWORDS: Conservation agriculture, soybean, weed management, nutrient dynamics, Vertisols.

---

*__Corresponding author__. Present address: Division of Soil chemistry and fertility, ICAR-Indian Institute of Soil Science, Nabibagh, Bhopal (Madhya Pradesh) 462 038, India.

E-mail address**:** bharatmeena24@gmail.com (Bharat Prakash Meena).

INTRODUCTION

The global population is growing at a faster rate and it has reached to 7.8 billion in 2020. It is estimated to grow further up to 8.5 billion by 2030, 9.7 billion by 2050 and to 10.9 billion by the year 2100 with present trends of population growth rate, [1, 2]. The food production in 2050 will need to increase by 50 percent as compared to 2012 to address the food grain demand. Achieving this projected demand and sustaining the production and productivity levels will be a major challenge in years to come [3]. Adoption of high-yielding varieties, fertilizer application, irrigation and plant protection will remain the most likely options to achieve intensification in agriculture to combat these challenges [4]. However, in recent years the global emphasis has shifted from improving potential yield levels to environmental concerns, soil health, reducing costs of production, better nutritional value, reduction in post-harvest losses, improving stress tolerance ability of crops and reducing dependency on chemical plant protection measures [5] with correspondence to climate change. Thus, sustainability of future agricultural systems, in the years to come will remain even greater challenge than the present scenario. Tillage has been considered as an essential component under conventional agriculture which makes soil friable, alters weed and nutrient dynamics to help crop establishment and improves physical properties of soil [6, 7]. However, the negative impact of tillage and other aspects in conventional agriculture based production techniques are hidden and are considered as necessary evils [8], However, it adversely affects soil by promoting soil erosion due to its destructive effect of tillage on soil structure and aggregation [9]. Extensive tillage coupled with crop residues burning has resulted in significant reduction in organic matter content and labile carbon pools of the soil [10, 11, 12], deterioration in soil physical properties [13] besides being capital- and energy-intensive, tillage accounts for

a substantial cost in the total cost of production leading to low economic returns [11]. CA has been considered as one of the most potential technology which has the potential to not only address the issues related to degradation of natural resource and environmental concerns but will also help in enhancing and sustaining the system productivity [14]. It is aimed at achieving production intensification and high yields together with improvement in the natural resource base by adopting resource-conserving sustainable agricultural production system. Farmers in India can adopt CA looking to multidimensional advantages of the technology through savings in cost of cultivation [6], improved quality of soil, *i.e.* physical, chemical and biological parameters [15, 16], build up of soil organic matter and increased C sequestration [17, 18, 19, 20], reduced infestation of weeds in long term [21] and improved input use efficiency particularly water and nutrients [22, 23].

Weed management ~~in~~ considered to be most daunting task under CA compared to conventional agriculture due to the fact that weed seed burial and destruction of vegetative structures by tillage operations are lacking in CA [24]. Compared to CT, more weed seeds are present on surface of the soil, which resulted in relatively higher accumulation and germination of more weeds under ZT. Hence, CA generally increases weed infestation during initial years due to reduction in tillage intensity [25]. The major hindrance in large scale adoption of CA based production systems is weed management and hence, it is necessary to assess the weed dynamics and their characteristics under CA for successful adoption on large scale [26, 27]. Reduction in crop productivity due to increased weed infestation and changes in relative densities of weeds under CA are other major concern restricting spread of CA [28]. Weed control under CA mainly rely on herbicidal weed management [29, 30]. Reduced tillage and greater dependency on herbicides for weed management under CA can bring substantial changes in weed dynamics and their populations [31, 32, 33]. Utilization of post-emergence broad-spectrum herbicides in CA based production systems may provide additional opportunity to tackle weed problem [34]. Proper weed management is necessary for obtaining comparable crop yields under conservation tillage system similar to that under conventional tillage system [35]. Thus, proper weed management needs special attention for successful adoption of CA on large scale. Application of sequential non-selective herbicides for weed control has paved the way for satisfactory crop establishment and consistent yield and creditable performance by providing season-long effective weed control and reducing chances of shift in weed flora towards more prolific weeds due to evolution of herbicide-resistant or tolerant biotypes of weeds [6, 36].

Soybean an important pulse crops in India cultivated during *Kharif* season for its multiple uses and is popular as an oilseed crop. India ranks fifth with 3.95% of global share in production among the soybean producing countries and occupies about 9.0% of the global acreage (120 m ha) under soybean cultivation. India has recorded a production (10.93 million tons) and productivity (1009 kg ha^{-1}) of soybean during the ~~*kharif* season~~ 2018 [37]. Among various pests, weeds are the most important and vigorous competitors of soybean which germinate and emerge with the crop, established ahead and soon cover the crop which is relatively short stature and susceptible to shading during its initial growth phase [38]. Thus, weeds constitute the most important yield limiting constraint in soybean based production systems acquiring a major portion of resources that are essential for plant growth and development [39, 40]. In general, weeds are competes for light, nutrients, and moisture during early growth stage with crops and pose early-season competition [41] and it is considered to be the most critical aspect early growth stage and needs major attention on management during this period. Depending upon the types and intensity of weeds crop- weed competition during critical period of crop growth results in 58 to 85%, reduction in yield of soybean [42, 43] and also increase production cost [44]. Manual weed control is exhaustive, labour intensive, difficult to perform in time during rainy season besides being expensive and therefore, limits the acreage [45]. Effective management of weeds is necessary to maintain productivity of agricultural crops [45]. Losses in soybean due to weed infestation is the major limiting factors responsible for low crop yields, even under conventional agriculture, with all tillage and herbicide usage. Due to rapid urbanization and industrialization labourers' availability at the critical period of crop weed competition (CWC) for weed management is becoming scarce and expensive during peak periods of demand [46, 47]. It has been estimated that the active population engaged in agriculture decreased from 64% in 1990 to 57% in 2005, thus restricting availability of economically available workforce in agriculture [48]. Among various pesticides used in agriculture, herbicides account for highest volume eclipsing all other major pesticide groups [49]. Vertisols, being rich in clay (>40%) content are problematic for intercultural operations because of excessive stickiness during *kharif* season, besides being not permissible in CA. Thus, weed management by applying herbicides is the only viable option for the success of CA in Vertisols [50]. However, among various herbicides there is no herbicide that can provide satisfactory control of different kinds of weeds present in CA field prior to sowing, as well as those which will emerge later and provide season long control.

Since, weeds are not controlled through tillage, it is expected that herbicides use control of weeds

under CA will increase [51]. Therefore, it is necessary to standardize effective strategy for control the weeds present before sowing of crop and to take care of weeds that will emerge during the course of crop growth in the presence of crop residue under CA. Under such circumstances, it may not be possible to achieve satisfactory weed control using single herbicide. Thus, it is advocated to use herbicide mixtures to improve the weed control spectrum under CA [24, 52, 53]. For proper weed control and satisfactory crop performance under zero till systems, utilization of proper dose and application of herbicide at right stage is necessary [54]. Since there is lack of scientific information on weed management aspect of conservation agriculture in Vertisols of central India, experiment was taken up to generate information on effective weed management technology for soybean crop through use of herbicides either alone, their sequential or combined application to improve weed control efficiency, sustaining crop productivity and environmentally safe practice for long term sustainable weed management under CA.

MATERIALS AND METHODS

Soil and climate

A field experiment was taken up with soybean crop under CA systems during *Kharif* season 2015 to 2017 at the Research Farm of ICAR-Indian Institute of Soil Science, Bhopal in Vertisols of Central India. The experimental site is located with latitude of 23º18´28.26” N, longitude of 77º24´26.00” E and 485 m above mean sea level. The soil of the experimental site was an *Isohyperthermic*, *Typichaplustert* and deep heavy clay in texture (47.2% clay, 30.5% silt, 22.3% sand), slightly alkaline (pH 8.2) in reaction having a bulk density 1.35 Mg m^{-3}, electrical conductivity (EC) 0.18dS m^{-1}, soil organic carbon (SOC) 0.68%, available nitrogen 266 kg ha^{-1}, phosphorus 27.34 kg ha^{-1} and potassium 524 kg ha^{-1} respectively. The decadal average rainfall in the experimental farm is 1146 mm, more than 80% of which occurs from June to September and potential evapo-transpiration of 1400 mm. The mean annual air temperature of experimental area is 25ºC. The climate in the experimental site is humid subtropical, with warm and humid monsoons during mid of June to end of September. The region has moderate to severe hot summer season from mid of March to mid-June with mean monthly maximum temperature of 41.8–46.2 ºC in May. In winter the temperature may occasionally drop close to freezing point and peaks in January when with mean minimum temperature of 9.7–11.8 ºC in January.

Conservation agriculture practices

Sowing was done with the help of ‘happy seeder’ at the onset of monsoon under no till system. A residue

level of 30% was maintained uniformly in all the plots irrespective of herbicidal treatments. Variety JS-335 of soybean was planted in the experimental field at a row spacing of 27.5 cm during the last week of June. Recommended rate of fertilizer (30 kg N+ 60 kg P_2O_5 +40 kg K_2O ha^{-1}) has been drilled uniformly at the time of sowing with 'happy seeder' as basal application. Pre-emergence application of herbicides was done immediately after sowing followed by post-emergence treatment was applied as per scheduled interval. The recommended package of agronomic practices and need based plant protection measures were adopted.

Treatment details and experimental set-up

The experiment was carried out in a randomized block design comprising of seven treatments and three replications (plot size=40 m^2). The treatment combinations (Table 1) were comprised *viz*., glyphosate has been applied as pre-emergence application either alone (T_4 and T_5) or in combination with pre-emergence herbicide pendimethalin (T_3, T_6 and T_7) for control of weeds already present in the field as per treatments except in hand weeding and control treatments where already emerged weeds present in the field prior to sowing were removed manually. Observations on plant growth, yield attributes, yield, weed population at different growth stages, crop and weed biomass at harvest were recorded. Observations on weed density were recorded with the help of a quadrant 0.25m^2 placed randomly at four places in each plot. The growth, yield attributes and yields were recorded from net plot area. Plant and seed samples were analysed to determine the nutrient uptake. Weed control efficiency (WCE) was calculated based on weed population and weed biomass generated at harvest stage using the following formula and expressed in percentage.

$$\mathrm{WCEp}\ (\%) = \frac{(\mathrm{WPC} - \mathrm{WPT})}{\mathrm{WPC}} \times 100$$

Where, WCEp: Weed control efficiency population; WPC: weeds population in control plot; and WPT: weed population in treated plot.

$$\mathrm{WCEb}\ (\%) = \frac{(\mathrm{DWC} - \mathrm{D\ WT})}{\mathrm{DWC}} \times 100$$

Where, WCEb: Weed control efficiency biomass; DWC: ~~dry weight~~ of weeds in control ~~Plot~~; and DWT: ~~dry weight~~ of weeds in treated plot.

The weed index (WI) was calculated for different treatments using the formula as suggested by Gill and Kumar [55].

$$\mathrm{WI}\ (\%) = \frac{(\mathrm{X} - \mathrm{Y})}{\mathrm{X}} \times 100$$

Where, X = Grain yield from weed free check, Y = Grain yield from treatment.

Table1. Details description of treatments used in the experiment during study.

Treatment	
T_1	Absolute control
T_2	Two hand weedings at 20 and 40 DAS
T_3	Pre-emergence application of pendimethalin @ 1kg ai ha^{-1} + Glyphosate @1 kg ai ha^{-1}
T_4	Pre-emergence application of Glyphosate @1 kg ai ha^{-1} followed by Post- emergence application of Propaquizafop @100g + Chlorimuron ethyl @ 9 g ai ha^{-1} at 20 DAS
T_5	Pre-emergence application of Glyphosate @ 1 kg ai ha^{-1} followed by Post- emergence application of Imazethapyr @100 g ai ha^{-1} at 20 DAS
T_6	T_3 followed by post- emergence application of Imazethapyr @ 100 g ai ha^{-1} at 30 DAS
T_7	T_3 followed by post- emergence application of Propaquizafop @ 100 g + Chlorimuron ethyl @ 9 g ai ha^{-1} at 30 DAS.

#Rate of dilution is 500 l ha^{-1}; DAS, days after sowing

Collection and analysis of plant samples

Ten random samples of soybean plants and weed samples from quadrats were drawn from each treatment at the time of harvesting, the samples were air-dried, and kept in an oven at 65°C until constant weight was obtained. The sample was powdered with the help of a grinder and these samples were used for the determination of nutrient and carbon content. Wet oxidation technique as described by Jackson [56] was adopted for determination of total C content in the samples. Total nitrogen in the crop residues were determined by micro Kjeldhal method after digesting in concentrated sulphuric acid [57]. P and K were determined by digesting the samples in a mixture of HNO_3 and $HClO_4$ (9:4) as suggested by Singh *et al.* [58]. The total P was determined by vanado molybdate yellow colour method as described by Jackson [59] whereas total K was determined by using flame photometer technique [57] in plant samples.

Statistical Analysis

The 3 years field experiment data on soybean crop and weeds were statistically analysed using SAS 9.3. The square root ($\sqrt{}$ (X + 0.5)) transformation was applied on weed density for holding the normality and independence assumptions. Combined/pooled analysis of variance (ANOVA) was carried out through PROC GLM. The treatments mean comparison for different parameters was performed based on Tukey's Honest Significant Difference (HSD) at P = 0.05 through MEANS statement under PROC GLM. Correlation

study was done for understanding the relationship between the parameters by using PROC CORR. Linear bivariate regression analysis was performed between crop yield with ~~weed control efficiency~~ and nutrients uptake using PROC REG. The multiple linear regression model was fitted for grain yield by using stepwise selection under PROC REG.

RESULTS

Weed flora and weed density

The major weed flora includes monocot weeds like *Echinochloa colonum* (L.) Link., *Dichanthium annulatum* forssk, *Brachiaria plantaginea* Griseb and *Commelina benghalensis* L. and among dicot weeds, *Acalypha indica* L. *Euphorbia geniculata* L., *Phyllanthus niruri* L., *Euphorbia hirta* L., *Digera arvensis Forsk.*, *Alternanthera sessilis* (L.), *Parthenium hysterophorus* L., *Celosia argentea* L., *Caesulia axillaris* Roxb. and *Cyanotis ciliaris* (L.). However, *Cyprus rotundas* (L). was the only weed belonging to sedges category, besides this few minor weeds were also present in the plot under CA. The weed flora was dominated by monocot weeds in the experimental field and recorded 62.3 % density as against dicot weeds which contribute of the extent of 35% and sedges 2.6%. The most dominant weed among different weed species was *Echinochloa colonum* (L.) Link. with a density of 106.64 m^{-2} sharing 33.70% of total weed population while *Dichanthium annulatum* forssk with a density of 70.64m^{-2} with 22.32% share was second followed by *Acalypha indica* L. (44 plants m^{-2}) and relative density of 13.9%. Data pertaining to weed density at different growth stages under various treatments (Table 2) revealed that there was significant effect of different weed management practices under CA. Application of glyphosate alone and in combination with pendimethalin as pre-emergence application resulted in control of already emerged weed in treatments T3, T6 and T7. Weed density at 20 days after sowing (DAS) showed that it was on par with respect to T1, T2, T4 and T5 indicating uniformity in weed density among different treatments prior to application of treatment *viz.*, hand weeding and post-emergence application of herbicidal weed control treatments. However, Treatments T3, T6 and T7 recorded significantly lower values of weed density (5.15, 6.47 and 5.3[illegible]) as compared to other treatments at 20 DAS. The significant lower values of weed density in treatments (T3, T6 and T7) indicate reduction in weed density due to pre-emergence application of pendimethalin in these treatments, which were on par with each other. At 60 DAS and at harvest stage all the weed control treatments recorded significantly lower weed densities as compared to T1 (No weeding control) (14.44and 17.73). Among weed control treatments, treatment T7 recorded lowest weed density

(8.95) at harvest stage which was at par with other weed control treatments except T4.

Table 2. Effect of different weed management practices on weed densities at different growth stages in soybean.

Treatment	Weed density (numbers m^{-2})					
	20 DAS		60 DAS		At harvest	
T_1	12.60^{a}	(165.1)	14.44^{a}	(209.1)	17.73^{a}	(316.44)
T_2	12.54^{a}	(156.9)	4.59^{bc}	(20.9)	9.85^{b}	(108.00)
T_3	5.15^{b}	(24.7)	8.19^{b}	(67.1)	11.03^{b}	(122.89)
T_4	12.34^{a}	(150.3)	6.79^{bc}	(48.5)	11.83^{ab}	(140.54)
T_5	12.19^{a}	(152.9)	6.86^{bc}	(50.0)	11.23^{b}	(127.11)
T_6	6.47^{b}	(36.5)	4.67^{bc}	(22.1)	9.13^{b}	(88.44)
T_7	5.36^{b}	(26.8)	4.04^{c}	(16.3)	8.95^{b}	(81.78)

Note: Weed data subject to $\sqrt{X + 0.5}$ transformation. Figures in parenthesis are actual values.
Pooled means followed by the same letters are not significantly different according to Tukey's Honest Significant Difference (P=0.05).

Weed control efficiency, weed biomass and weed index

Among different treatments, maximum weed control efficiency in terms of population at harvest stage (72.83%) has been recorded under T7 (Table 3) followed by T6 (70.66%). Better weed control under herbicide applied plots were attributed to better weed control and improved crop performance due to sequential application of herbicides in combination of pendimethalin + glyphosate as pre-emergence followed by post emergence application of propaquizafop+ chlorimuron ethyl (T7) or imazethapyr (T6) at 30 DAS. Among different treatments, lowest weed biomass (562kg ha^{-1}) was recorded under T2 (Hand weeding) which was statistically at par with treatment T7 (814 kg ha^{-1}). Maximum weed biomass 3850 kg ha^{-1} was recorded (Table 3) under control (T1). Maximum weed control efficiency (WCI) in terms of biomass (81.95%) has been recorded under T2 followed by treatment T6 (81.52%). Lower weed index (0.32%) was recorded under treatment T6. Maximum weed index (74.55%) was recorded under control (T1), indicating the severity of weed infestation and competition under Vertisols which ultimately resulted in significant yield reduction under no weeding control (T1) treatment.

Table 3. Effect of different weed management practices on weed biomass, weed control efficiency (WCE) and weed index at harvest.

Treatment	Weed control efficiency Population (%)	Weed dry biomass (kg ha^{-1})	Weed control efficiency Biomass (%)	Weed index (%)
T_1	0.00	3848^{a}	0.00	75.86
T_2	66.61	562^{d}	81.95	0.00
T_3	59.16	1880^{b}	55.35	42.84
T_4	55.32	1758^{b}	55.77	22.86
T_5	59.70	1614^{bc}	61.82	12.95
T_6	70.66	814^{d}	81.52	0.32
T_7	72.83	1425^{c}	67.32	7.97

Pooled means followed by the same letters are not significantly different according to Tukey's Honest Significant Difference (P=0.05).

Growth and yields

Significant influence of various herbicidal weed control treatments on plant growth and yield parameters were recorded as a result of their impact on weed population and their growth as compared to no weeding control (T1). Maximum plant height (50.98 cm) was recorded under T2 which was at par with rest of the herbicidal weed control treatments except control ~~(T1)~~ (Table 4). Maximum number of branches per plant (8.60) was recorded under T2 which was at par with rest of the herbicidal weed control treatments. Lowest number of branches per plant (6.71) was recorded under control (T1). The no weeding control treatment recorded significantly lower number of pods per plant (17.23) as compared to other weed control treatments. Significantly higher number of pods per plant (43.43) was recorded under treatment T2 which was on par with treatments T6 and T7 with 41.08 and 39.98 pods per plant. Similarly all the weed control treatments produced higher number of seeds per pods as compared to control (2.17). Maximum number of seeds per pod (2.56) was recorded under treatment T2 which was at par with treatments T4, T6 and T7 and significantly higher as compared to rest of the weed control treatments. Maximum crop biomass and straw yield (4513 kgha^{-1} and 2951 kgha^{-1}) was recorded under treatment T6 which was on par with rest of the herbicidal weed control treatments and hand weeding and significantly superior over treatment T1 andT3. Maximum seed yield (1567 kg ha^{-1}) and harvest index (37.37%) was recorded under treatment T2 which was at par with treatments T6, T7 and T5 and significantly higher as compared to treatments T1, T3 and T4.

Table 4. Effect of different weed management practices on crop growth, yield attributes and yields.

Treatment	Plant height (cm)	Branches plant^{-1}	Pods plant^{-1}	Seeds pod^{-1}	Biological yield (kgha^{-1})	Straw yield (kg ha^{-1})	Grain yield (kg ha^{-1})	Harvest index (%)
T_1	38.90^{b}	6.71^{b}	17.23^{c}	2.17^{b}	1181^{c}	810^{c}	371^{c}	31.47^{a}
T_2	50.98^{a}	8.60^{a}	43.43^{a}	2.56^{a}	4437^{a}	2870^{a}	1567^{a}	35.37^{a}
T_3	45.39^{ab}	8.06^{a}	34.63^{b}	2.26^{b}	2946^{b}	2058^{b}	888^{b}	30.53^{a}
T_4	50.42^{a}	8.46^{a}	37.90^{ab}	2.35^{ab}	3660^{ab}	2463^{ab}	1196^{ab}	32.80^{a}
T_5	49.30^{a}	8.17^{a}	35.56^{b}	2.28^{b}	3853^{ab}	2503^{ab}	1350^{a}	35.07^{a}
T_6	49.89^{a}	8.57^{a}	41.08^{ab}	2.39^{ab}	4513^{a}	2951^{a}	1562^{a}	35.13^{a}
T_7	49.27^{a}	8.47^{a}	39.98^{ab}	2.36^{ab}	4236^{a}	2805^{ab}	1431^{a}	33.97^{a}

Pooled means followed by the same letters are not significantly different according to Tukey's Honest Significant Difference (P=0.05).

Nutrient uptake and nutrient use efficiency

Nutrient uptake in grain and straw was also found to be significantly influenced in response of different herbicidal treatments in soybean (Table 5). The highest total nutrient uptake (235.27 kg ha^{-1}) was recorded under T6 where sequential per-emergence and post-emergence herbicides was applied and followed by T2 (hand weedings at 20 and 40 DAS) (232.19 kg ha^{-1}) which was at par with T2 (Hand weedings only) T4, T5 and T7 treatments while lowest nutrient (NPK) uptake in soybean was noticed in control plots. Furthermore, the results revealed that pre-emergence of herbicides (T3) of also performed better in respect to nutrient uptake as compared to control plots.

Table 5. Effect of different weed management practices on nutrient (NPK) uptake in soybean

	Nutrient uptake Grain (kg ha^{-1})			Nutrient uptake straw (kg ha^{-1})			Total Nutrient uptake (kg ha^{-1})			
Treatment	N	P	K	N	P	K	N	P	K	NPK
T_1	17.16^{d}	1.72^{c}	5.79^{d}	20.43^{c}	2.87^{b}	9.93^{c}	37.60^{c}	4.60^{c}	15.72^{c}	57.94^{c}
T_2	76.10^{a}	7.52^{a}	25.46^{a}	77.10^{a}	9.38^{a}	36.61^{ab}	153.20^{a}	16.90^{a}	62.07^{a}	232.19^{a}
T_3	41.62^{c}	4.12^{b}	13.58^{c}	53.01^{b}	7.28^{a}	26.67^{b}	94.64^{b}	11.42^{b}	40.26^{b}	146.32^{b}
T_4	57.39^{bc}	5.82^{ab}	18.36^{bc}	68.03^{ab}	8.88^{a}	33.08^{ab}	125.43^{ab}	14.70^{ab}	51.44^{ab}	191.59^{ab}
T_5	65.37^{ab}	6.68^{a}	23.27^{ab}	67.80^{ab}	8.05^{a}	32.79^{ab}	133.17^{a}	14.74^{ab}	56.07^{a}	204.00^{a}
T_6	74.36^{ab}	7.41^{a}	26.26^{a}	80.59^{a}	9.34^{a}	37.29^{a}	154.95^{a}	16.75^{a}	63.55^{a}	235.27^{a}
T_7	67.66^{ab}	6.60^{a}	22.93^{ab}	77.04^{a}	8.85^{a}	36.03^{ab}	144.90^{a}	15.45^{a}	58.97^{a}	234.19^{a}

Pooled means followed by the same letters are not significantly different according to Tukey's Honest Significant Difference (P=0.05).

Agronomic use efficiency (AUE) for N, P and K also significantly increased under different treatments whereas physiological use efficiency (PUE) (Table 6) did not significantly influenced by the applied herbicidal weed management practices in soybean. The highest agronomic use efficiency for nitrogen (52.23 kg $kg^{-1}$of nutrient applied) was recorded in T2 treatment followed by T6 (52.09 kg $kg^{-1}$of nutrient applied), T7 (547.70 kg $kg^{-1}$of nutrient applied) and T5 (45.01) kg $kg^{-1}$of nutrient applied) and similar trend was also followed in case of P and K. While, the lowest agronomic use efficiency (AUE) was noticed in control plots in respect to N, P and K.

Table 6. Agronomic and physiological efficiencies influenced by weed management practices in soybean.

Treatment	Agronomic use efficiency (kg kg^{-1} of nutrient applied)			Physiological use efficiency (kg kg^{-1} of nutrient uptake)		
	N	P	K	N	P	K
T_1	12.37^{c}	6.18^{c}	9.27^{c}	$9.\ 87^{a}$	80.63^{a}	23.62^{a}
T_2	52.23^{a}	26.11^{a}	39.17^{a}	10.22^{a}	93.23^{a}	25.31^{a}
T_3	29.60^{b}	14.80^{b}	22.20^{b}	9.46^{a}	78.86^{a}	22.41^{a}
T_4	39.89^{ab}	19.94^{ab}	29.92^{ab}	9.56^{a}	81.68^{a}	23.33^{a}
T_5	45.01^{a}	22.50^{a}	33.76^{a}	10.12^{a}	91.60^{a}	24.07^{a}
T_6	52.09^{a}	26.04^{a}	39.07^{a}	10.18^{a}	94.55^{a}	24.89^{a}
T_7	47.70^{a}	23.85^{a}	35.77^{a}	9.90^{a}	93.27^{a}	24.37^{a}

Pooled means followed by the same letters are not significantly different according to Tukey's Honest Significant Difference (P=0.05).

Crop residue retention, carbon and nutrient recycling

As a result of better crop performance in terms of crop growth, more crop biomass, carbon and nutrient accumulation has been observed. Residue retention, carbon input and nutrient recycled in soybean was also significantly influenced under different herbicidal weed management treatments under CA, highest residue (885 kg $ha^{-1}year^{-1}$), carbon input (354 kg $ha^{-1}year^{-1}$), and nutrient recycled (24.17, 2.80 and 11.18 kg N, P and K $ha^{-1}year^{-1}$) was recorded under treatment T6 which was at par with treatment T2, T7, T5 and T4 and significantly higher as compared to treatmentsT1 and T3 during the course of study (Table 7).

Table 7. Carbon and nutrient addition through residue recycling in soybean

Treatment	Residue recycled (kg ha^{-1}year^{-1})	Carbon input (kg ha^{-1} year^{-1})	Nutrient recycled (kg ha^{-1} year^{-1})		
			N	P	K
T_1	243^{c}	97^{c}	6.13^{c}	0.86^{b}	2.97^{c}
T_2	861^{a}	344^{a}	23.13^{a}	2.81^{a}	10.98^{ab}
T_3	617^{b}	246^{b}	15.90^{b}	2.18^{a}	8.00^{b}
T_4	739^{ab}	295^{ab}	20.41^{ab}	2.66^{a}	9.92^{ab}
T_5	751^{ab}	300^{ab}	20.34^{ab}	2.41^{a}	9.84^{ab}
T_6	885^{a}	354^{a}	24.17^{a}	2.80^{a}	11.18^{a}
T_7	841^{ab}	336^{ab}	23.11^{a}	2.65^{a}	10.81^{ab}

Pooled means followed by the same letters are not significantly different according to Tukey's Honest Significant Difference (P=0.05).

Correlation and regression relationship to yield and weed parameters

Pearson's correlation coefficients matrix (Table 8) revealed that grain yield was significantly (P<0.0001) related to WD60 (r=-0.84), WDH (r=-0.63), WDB (r=-0.52), WN (r=-0.56), WP (r=-0.59), WK (r=-0.66) and WI (r=-0.96). The bivariate linear regression study of grain yield on ~~weed control efficiency~~ biomass, N, P and K uptake showed the clear significant (P<0.0001) dependence with R^2 value of 0.53 (Fig. 1), 0.99 (Fig. 2), 0.95 (Fig. 3) and 0.98 (Fig. 4), respectively.

Fig1. Relationship between grain yield (GY) and weed control efficiency biomass (WCEB) under herbicidal weed management practices in soybean.

Fig 2. Relationship between grain yield (GY) and N Uptake in grain (NG) under herbicidal weed management practices in soybean.

Fig 3. Relationship between grain yield (GY) and P Uptake in grain (PG) under herbicidal weed management practices in soybean.

Fig 4. Relationship between grain yield (GY) with K Uptake in grain (KG) under herbicidal weed management practices in soybean.

The stepwise multiple regression (model) analysis (Eq. 1), indicated that some variables has truly influenced (significantly) the grain yield as compared to others. The fitted model showed that among the different

parameters, only N and P uptake in grain, weed density at 20 DAS and K uptake in weed were actual predictor for grain yield.

GY = 0.19**xNG+0.21**xPG-0.04**xWD20+0.01*xWK, (R^2 = 0.99; P<0.0001) (Eq. 1)

(*and** indicate the parameters estimate are significant at P=0.05 and P=0.01)

Table 8. Pearson's correlation coefficients (N=63) between yield and weed parameters

Parameters	GY	WD20	WD60	WDH	WDB	WN	WP	WK	WI
**GY**	1.00000	-0.12846 (0.3157)	-0.84432 (<.0001)	-0.63054 (<.0001)	-0.51733 (<.0001)	-0.55532 (<.0001)	-0.59068 (<.0001)	-0.65661 (<.0001)	-0.96115 (<.0001)
**WD20**		1.00000	0.36129 (0.0036)	0.48020 (<.0001)	0.26826 (0.0335)	0.28162 (0.0254)	0.34194 (0.0061)	0.28395 (0.0241)	0.13302 (0.2987)
**WD60**			1.00000	0.78447 (<.0001)	0.63483 (<.0001)	0.66898 (<.0001)	0.71506 (<.0001)	0.76218 (<.0001)	0.81590 (<.0001)
**WDH**				1.00000	0.62353 (<.0001)	0.64794 (<.0001)	0.69424 (<.0001)	0.71356 (<.0001)	0.65138 (<.0001)
**WDB**					1.00000	0.99688 (<.0001)	0.98677 (<.0001)	0.96859 (<.0001)	0.52629 (<.0001)
**WN**						1.00000	0.98894 (<.0001)	0.97746 (<.0001)	0.56251 (<.0001)
**WP**							1.00000	0.98015 (<.0001)	0.59566 (<.0001)
**WK**								1.00000	0.65902 (<.0001)
**WI**									1.00000

GY, Grain yield; WD20, Weed density at 20 DAS; WD60, Weed density at 60 DAS; WDH, Weed density at harvest; WDB, Weed dry biomass; WN, N uptake in weed; WP, P uptake in weed; WK, K uptake in weed; WI, Weed index

Values in the parenthesis is the actual probability level of significance; if the value is ≤0.05 or 0.01, then r (the correlation coefficient) ≠ 0 at P=0.05 or 0.01.

DISCUSSION

Weed flora and weed density

In the experimental field monocot weeds were dominant and recorded 62.3 % density as compared of dicot weeds which contribute to the extent of 35% and sedges 2.6%. Among monocot weed species *Echinochloa colonum* ([illegible]) was the most dominant weed followed by *Dichanthium annulatum* forssk. Among dicot weeds, *Acalypha indica* L. was the dominant one. Similar, weed flora in soybean cultivated in Vertisols were also reported by Kewat *et al*. [42] and Kalhapure *et al*. [60]. Significant effect of different weed control treatme[illegible]n density of dominant weeds at different growth stages was recorded under CA (Table 2).

Application of glyphosate alone and in combination with pendimethalin as pre-emergence application has effectively controlled emerged weeds as well as do not allow fresh flush during initial crop growth phase [61] and also reported significant reduction in weed density of all the species due to pre-emergence application of herbicides as compared to weedy check. Significantly lower weed densities in all the treatments as compared to no weeding control may be attributed to the action of different combinations of pre- and post-emergence herbicide treatments applied to soybean crop, whereas in case of pendimethalin alone treatment the number of weeds increased substantially towards maturity of crop. Application of pendimethalin resulted in inhibition of microtubule formation in cells of sensitive weeds, thus interfere with the cell division process of germinating seeds. This resulted in restricted cell division and reduced growth of the emerging weed seedling eventuated to lack of food reserves and ultimately results in death of germinating seedlings. Similar results of application of pendimethalin in soybean were also reported by Malik *et al*. [62]. Inhibition of major enzymatic processes responsible for protein synthesis *viz*., acetolactate synthase (ALS) or acetohydroxy acid synthase (AHAS) in broadleaf weeds due to post-emergence application of imazethapyr at 3–4 leaf stage is responsible for control of these weeds due to starvation [63]. Propaquizafop a selective grass killer herbicide used for controlling grassy weeds in broadleaf crops kills grasses by disrupting the function of acetyl-CoA carboxylase (ACCase) mediated processes by binding it to the target site resulting in disruption of protein synthesis in the weeds which is involved in the production of oils needed for plant growth. Chlorimuron ethyl inhibits activity of actetolactate synthase (ALS) enzyme, which is involved in synthesis of many essential branched chain amino acids including valine, leucine, and isoleucine. Inhibition of ALS enzyme mediated processes resulted in gradual killing of target weeds in mixed population. Similar findings were also reported by Kumar *et al*. [64]. Lowest weed dry matter, weed index and highest ~~weed control efficiency~~ was found in treatment T7 involving pre-emergence application of pendimethalin at 1 kg ai ha^{-1} + glyphosate at 1 kg ai ha^{-1} followed by imazethapyr 0.100 kg ai ha^{-1} as PoE. This may be attributed mainly to better weed control as a result of post-emergence application of imazethapyr applied at 3–4 leaf stage [63, 65].

Weed control efficiency, weed biomass and weed index

Season long weed control and higher ~~weed control efficiency~~ in terms of population due to sequential application of pre- and post-emergence herbicides gave better results as compared to their individual applications. Thus, resulted in lower weed biomass, improved crop performance and lower weed index

under these treatments [24] also reported greater efficacy at lower cost with post-emergence application of herbicides and these findings were also in close conformity of the same. Higher ~~weed control efficiency~~ (WCI) underhand weeding treatment in terms of biomass as compared to WCI in terms of population may be attributed to the fact that a higher number of weeds emerged towards maturity phase of the crop which could not attain higher biomass as compared to weeds which emerge earlier during the crop cycle in other treatments. These results were in close conformity with that of reported by Mishra and Singh [61]. As a result of increased crop yield under different weed control treatments, lower weed index was observed in treated plots which indicate the severity of weed infestation and competition by weeds in soybean under Vertisols which resulted in significant yield reduction under no weeding control treatment.

Growth and yields

Significant response of various herbicidal treatments on plant growth and yield parameters of soybean may be attributed to availability of favourable environment for crop as a result of proper weed control, which reduces the competition of crop with weeds for space, air, sunlight, moisture and nutrients which is reflected in terms of significantly higher seed yield, (1567 kg ha^{-1}) and harvest index (37.37%). Prachand *et al*. [65] and Singh *et al*. [66] also reported increased yield and harvest index in soybean as a result of weed management due to better crop growth and yield parameters. Muoni *et al*. [67] suggested that yield losses due to crop-weed competition are reduced as a result of decrease in weed density. Besides reducing the weed population and growth the herbicidal weed control treatments also resulted in reduced the nutrient depletion by weeds and thereby increasing the nutrient uptake by crop by providing congenial environment for crop growth and development which helped the soybean crop to produce a greater number of pods $plant^{-1}$ and seeds $plant^{-1}$. The improved growth and yield parameters ultimately reflected in terms of higher crop yield in herbicidal weed control treatments as compared to no weeding control and were at par with hand weeding treatment [68, 69, 70] also reported improvement in yield and economical parameters as a result of better weed control with herbicidal weed management practices in soybean.

Nutrient uptake and nutrient use efficiency

Weeds are more efficient competitors for nutrients, water, space utilization as compared to soybean, besides harbouring pest and diseases that affect crop growth and development, which ultimately have negative effects on crop yield and nutrient uptake by crop [71]. Significant response of different herbicidal treatments on nutrient uptake in seed and straw as compared to nutrient uptake in seed and straw under control was

recorded in treatments (Table 5). This might be due to better crop growth which resulted in higher grain and straw yield in the respective treatments and ultimately resulted in higher nutrient uptake due to availability of proper space and light to crop as a result of increased weed control in herbicidal treatments during critical periods of crop growth. This may also be attributed to the impact of lower competition under herbicide applied plots, which as a result of better weed suppression resulted in improved canopy development and increased the nutrient uptake. Mishra and Singh [61] and Wang *et al.* [72] also reported improved nutrient uptake as result of weed control treatments in soybean crop. Increase in nutrient uptake (NPK) also suggests that nutrient use efficiency was high under the given treatment due to reduced crop-weed competition and also increase the crop yield due to less weed population [67]. Significantly higher agronomic use efficiency (Table 6) were recorded under various weed control treatments, this may be attributed to the impact of weed control treatments in terms of better crop growth and reduced crop weed competition, which resulted in greater nutrient uptake under these treatments as compared to control. On the contrary physiological use efficiency did not influenced by the applied herbicidal weed management in soybean crops during the experimentation. Chauhan *et al*. [24] also reported similar observations and suggested that herbicidal weed management practices under conservation agriculture are very much suitable for obtaining higher nutrient use efficiency and higher yield. However, physiological use efficiencies of N, P and K were non-significant indicating that the biomass production is influenced by the amount of nutrients absorbed by soybean crop, however, the composition of biomass does not influenced significantly by the amount of nutrients absorbed by the crop.

Crop residue retention, carbon and nutrient recycling

Crop residue retention is core principle of conservation agricultural production system through its influence on the soil-plant inter-relationship, which not only helps in nutrient recycling in long term [73, 74], but also helps in soil health improvement [75] and reduction in soil degradation [76]. Significant influence of herbicidal weed management treatments in terms of crop biomass production, carbon and nutrient recycling under CA is due to retention of higher residue biomass under different treatments due to differential crop growth, which resulted in accumulation of more crop biomass, carbon and nutrient under different treatments [77]. Therefore, retention of higher amount of residue in treatments producing more residues as a result of better growth resulted in accumulation of higher amount of carbon in soybean based agricultural production systems and increased nutrient recycling. As a result of improved crop performance; the crop

acquires a major portion of resources that are required for growth and development of plant which ultimately resulted in increased residue, carbon and nutrient recycling [39, 40].

Correlation and regression relationship to yield and weed parameters

The correlation matrix represents the interrelationships among different variables, to which the crop yield is negatively correlated with all other yield affecting parameters *viz.*, density of the weeds, weed biomass, weed nutrient uptakes and weed index (Table 8). Pearson's correlation coefficients matrix revealed that grain yield was significantly ($P<0.0001$) related to all the parameters except weed density at 20 DAS. The bivariate linear regression study of grain yield on weed control efficiency (biomass), nutrient uptake (N, P and K) in grain showed the clear significant ($P<0.0001$) dependence with good fit R^2 values. Stepwise multiple regression analysis (Eq. 1) showed that N and P uptake in grain, weed density at 20 DAS and K uptake in weed was true significant predictor for grain yield.

CONCLUSION

Based on our findings we concluded that amongst various biotic constraints for crop production under CA weeds are the major one, and greatest challenge limiting its wide spread adoption in the farmer's field in Vertisols despite several advantages. Adoption of no tillage leads to increased weed infestation under CA due to predominance and accumulation of weed seeds on the upper surface of soil. Reliance on herbicidal weed management is the only feasible way to deal with this problem under CA. However, use of single herbicide may not be sufficient to provide season long control of wide spectrum of weeds. Thus, we concluded that under CA based soybean production system weeds can be effectively managed through utilization of different herbicides in suitable combination applied at desired rate at right time. Weeds, under soybean-based CA system pre-emergence application of pendimethalin at 1000 g ai ha^{-1}+ glyphosate at 1000 g ai ha^{-1} followed by post-emergence application of imazethapyr at 100 g ai ha^{-1} at 30 DAS which was on par with hand weeding and proved to be economically beneficial and practically feasible under the CA system in Vertisol.

DISCLOSURE STATEMENT

The authors declared that there is no conflict of interest among the authors.

ACKNOWLEDGMENT

Authors acknowledge ICAR-Indian Institute of Soil Science, Bhopa[illegible]dia for providing financial and technical assistance during the course of study.

REFERENCES

1. World population prospects. Population Data, File: Population Growth Rate, Medium Variant Fertility tab". United Nations Population Division. 2019.
2. Godfray HCJ, Beddington JR, Crute IR, Haddad L, Lawrence D, Muir JF, Pretty J, Robinson S, Thomas SM, Toulmin C. Food security: The challenge of feeding 9 billion people. Science. 2010; 327:812–818. doi: 10.1126/science.1185383.
3. FAO. The future of food and agriculture – Trends and challenges. Rome. 2017; pp. 1-163.
4. Chakraborty S, Chakraborty SK. "New and Advanced Technology in Today's Agriculture". EC Agriculture. 2016; 3.1: 566-569.
5. Brodt S, Six J, Feenstra G, Ingels C, Campbell D. Sustainable Agriculture. Nature Education Knowledge. 2011; 3(10): 1.
6. Malik RK, Gupta RK, Singh CM, Yadav A, Brar SS, Thakur TC, Singh SS, Singh AK, Singh R, Sinha RK. Accelerating the Adoption of Resource Conservation Technologies in Rice Wheat System of the Indo-Gangetic Plains. Proceedings of Project Workshop, Directorate of Extension Education, Chaudhary Charan Singh Haryana Agricultural University (CCSHAU), Hisar, India. June 1-2, 2005.
7. Bobby [illegible]tewart, A[illegible]ares Asfary[illegible]Abdelouahab Belloum, Kurt Steiner, Theodor Friedrich. Conservation Agriculture for Sustainable Land Management to Improve the Livelihood of People in Dry Areas. Damascus, Syria. 2008. http://www.fao.org/ag/ca/doc/CA%20Workshop%20procedding%2008-08-08.pdf.
8. Capellesso Adinor Jose, Ademir Antonio Cazella, Abdon Luiz Schmitt Filho, Joshua Farley, Diego Albino Martins. Economic and environmental impacts of production intensification in agriculture: comparing transgenic, conventional, and agro ecological maize crops. Agroecology and Sustainable Food Systems. 2016; 40(3): 215-236.
9. Zheng H, Liu W, Zheng J, Luo Y, Li R, Wang H. Effect of long-term tillage on soil aggregates and aggregate-associated carbon in black soil of Northeast China. PLOS ONE. 2018; 13(6), e0199523.

https://doi.org/10.1371/journal.pone.019952.

10. Bhattacharyya R, Prakash V, Kundu S, Srivastva AK, Gupta HS. Soil aggregation and organic matter in a sandy clay loam soil of the Indian Himalayas under different tillage and crop regimes. Agriculture, Ecosystems & Environment. 2009; 132:126–134.
11. Das TK. Weeds and their control methods. In: Weed management. 2014; pp. 9–14. http://www.nsdl.niscair.res.in/bitstream/123456789/568/1/Weeds+and+their+control+methods++Formatted.pdf. Accessed 6 March 2014.
12. Meena BP, Shirale AO, Dotaniya ML, Jha P, Meena AL, Biswas AK, Patra AK. Conservation Agriculture: A New Paradigm for Improving Input Use Efficiency and Crop Productivity. In: Bisht, J., Meena, V., Mishra, P., Pattanayak, A. (eds) Conservation Agriculture. Springer, Singapore. 2016; pp. 39-69. https://doi.org/10.1007/978-981-10-2558-7_2.
13. Mishra JS, Singh VP. Tillage and weed control effects on productivity of a dry seeded rice – wheat system on a vertisol in central India. Soil and Tillage Research. 2012; 123: 11–20.
14. Abrol IP, Sangar S. Sustaining Indian agriculture-conservation agriculture the way forward. Current Science. 2006; 91(8): 1020-2015.
15. Jat ML, Gathala MK, Ladha JK, Sehrawat IS, Jat AS, Kumar Vipin, Sharma SK, Kumar V, Gupta Raj. Evaluation of precision land leveling and double zero-till systems in rice wheat rotation: water use, productivity, profitability and soil physical properties. Soil and Tillage Research. 2009; 105: 112-121.
16. Kaschuk G, Alberton O, Hungria M. Three decades of soil microbial biomass studies in Brazilian ecosystems: lessons learned about soil quality and indications for improving sustainability. Soil Biology and Biochemistry. 2010; 42: 1–13.
17. Blanco Canqui H, Lal R. Crop residue removal impacts on soil productivity and environmental quality. Critical Reviews in Plant Sciences. 2009; 28: 139–16.
18. Kumawat A, Vishwakarma AK, Wanjari RH, Sharma NK, Yadav D. Impact of levels of residue retention on soil properties under conservation agriculture in Vertisols of central India. Archives of Agronomy and Soil Science. 2020. DOI: 10.1080/03650340.2020.1836345.
19. Meena BP, Biswas AK, Singh M, Das H, Chaudhary RS, Singh AB, Shirale AO, Patra AK. Energy budgeting and carbon footprint in long-term integrated nutrient management modules in a cereal- legume (Zea m[illegible]ys – Cicer arietinum) cropping system. Journal of Cleaner Production. 2021; 314: 127900.

https://doi.org/10.1016/j.jclepro.2021.127900.

20. Yadav Devideen, Vishwakarma AK, Sharma NK, Biswas AK, Ojasvi PR, Kumar Dinesh, Kumawat Anita, Singh Deepak. Sustaining the properties of black soil in Central India through crop residue management in a conservation-agriculture-based soybean–wheat system. Land Degradation & Development. 2021; 32(10): 2906-2921. DOI: 10.1002/ldr.3891.
21. Chauhan BS, Gill GS, Preston C.Effect of seeding systems and dinitroaniline herbicides on emergence and control of rigid ryegrass (Lolium rigidum) in wheat. Weed Technology. 2007; 21: 53–58.
22. Sehrawat IS, Ladha JK, Pathak H, Gathala M, Chaudhary N, Jat ML. Simulation of resource-conserving technologies on productivity, income and greenhouse gas emission in rice– wheat system. Journal of Soil Science and Environmental Management. 2012; 3: 9–22.
23. Jat HS, Datta A, Sharma PC, Kumar V, Yadav AK, Choudhary M, McDonald A. Assessing soil properties and nutrient availability under conservation agriculture practices in a reclaimed sodic soil in cereal-based systems of North-West India. Archives of Agronomy and Soil Science. 2018; 64: 531–545. https://doi.org/10.1080/03650340.2017.1359415.
24. Chauhan BS, Singh RG, Mahajan G. Ecology and management of weeds under conservation agriculture: a review. Crop Protection. 2012; 38: 57–65.
25. Singh VP, Barman KK, Singh Raghwendra, Sharma AR. Weed Management in Conservation Agriculture Systems in book entitled "Conservation Agriculture", M. Farooq, K. H. M. Siddique (eds.). 2015; pp. 39-77. DOI 10.1007/978-3-319-11620-4_3.
26. Lafond GP, McConkey BG, Stumborg M. Conservation tillage models for small-scale farming: linking the Canadian experience to the small farms of Inner Mongolia autonomous region in China. Soil and Tillage Research. 2009; 104: 150–15.
27. Giller KE, Witter E, Corbeels M, Tittonell P. Conservation agriculture and smallholder farming in Africa: the heretics view. Field Crops Research. 2009; 114: 23–34
28. Blackshaw RE, Larney FJ, Lindwall CW, Watson PR, Derksen DA. Tillage intensity and crop rotation affect weed community dynamics in a winter wheat cropping system. Canadian Journal of Plant Science. 2001; 81: 805-813.
29. Nicole Lee, Christian Thierfelder. Weed control under conservation agriculture in dryland smallholder farming systems of southern Africa: A review. Agronomy for Sustainable Development. 2017; 37: 48.

DOI: 10.1007/s13593-017-0453-7.

30. Vishwakarma AK, Wanjari RH, Brajendra, Ram Gopal. Weed management in conservation agriculture: A brief review. Journal of Pharmacognosy and Phytochemistry. 2017; SP1: 502-506.
31. Buhler DD, Stoltenberg DE, Becker RL, Gunsolus JL. Perennial weed populations after 14 years of variable tillage and cropping practices. Weed Science. 1994; 34: 29-33.
32. Virginia Nichols, Nele Verhulst, Rachael Cox, Bram Govaerts. Weed dynamics and conservation agriculture principles: A review. Field Crops Research. 2015; 183: 56-68. doi.org/10.1016/j.fcr.2015.07.012.
33. Bhagirath S. Chauhan, Gurjeet Gill C. Preston. Tillage system effects on weed ecology, herbicide activity and persistence: A review. Australian Journal of Experimental Agriculture. 2006; 46(12). DOI:10.1071/EA05291.
34. Nalewaja J. Weedsandconservation agriculture. World congress on conservation agriculture, Madrid. 2001; pp.191–200.
35. Mahajan G, Brar LS, Walia US. Phalarisminor response in wheat in relation to planting dates, tillage and herbicides. Indian Journal of Weed Science. 2002; 34: 213–215.
36. Akbar Hossain, Mst Tanjina Islam, Md Shohidul Islam, Nurislam Sharif Ahmed, Khokan Kumer Sarker, Mahesh Kumar Gathala. Chemical Weed Management in Maize under Conservation Agricultural Systems: An Outlook of the Eastern Gangetic Plains in South-Asia. 2019. DOI: 10.5772/intechopen.89030. Available from: https://www.intechopen.com/books/maize-production-and-use/chemical-weed-management-in-maize-em-zea-mays-em-l-under-conservation-agricultural-systems-an-outlook.
37. SOPA. http://www.sopa.org/crop (The Soybean Processors Association of India, SOPA). 2018.
38. Alexandre Ferreira da Silva, Leandro Galon, Ignacio Aspiazú, Evander Alves Ferreira, Germani Concenço, Edison Ulisses Ramos Júnior, Paulo Roberto Ribeiro Rocha. Weed Management in the Soybean Crop, Soybean - Pest Resistance, Hany A. El-Shemy, Intech Open. 2013. DOI: 10.5772/54596. Available from: https://www.intechopen.com/books/soybean-pest-resistance/weed-management-in-the-soybean-crop.
39. Ryan J, Singh M, Pala M. Long-term cereal based rotation experiments in the Mediterranean region: implications for cropping sustainability. Advances in Agronomy. 2008; 97: 273–318.

40. Smith P, Olesen JE. Synergies between the mitigation of, and adaptation to, climate change in agricultur e. Journal of Agricultural Science. 2010; 148: 543–552.
41. Shrestha A, Knezevic S, Roy R. Effect of tillage, cover crop and crop rotation on the composition of weed flora in a sandy soil. Weed Research. 2002; 42(1): 76-87.
42. Kewat ML, Pandey J, Yaduraju NT, Kulshreshtha G. Economic and ecofriendly weed management in soybean. Indian J. Weed Sci. 2000; 32(3 and 4): 135-139.
43. Tiwari JP, Khurchania SP. Survey and management of weed in soybean [Glycine max (L.) Merrill] ecosystem in M.P. Indian Journal of Agricultural Science. 1990; 60(10): 672-676.
44. Sharma AR, Jat ML, Saharawat YS, Singh VP, Singh R. Conservation agriculture for improving product ivity and resourceuse efficiency: prospects and research needs in Indian context. Indian Journal of Agronomy. 2012; 57 (3rd IAC Special Issue): 131–140.
45. Verma SK, Singh SB, Meena RN, Prasad SK, Meena RS, Gaurav. A review of weed management in India: The need of new directions for sustainable agriculture. The Bioscan. 2015; 10(1): 253-263.
46. Abouziena HF, Haggag WM. Weed Control in Clean Agriculture: A Review. Planta Daninha. 2016; 34(2): 377-392.
47. Nagargade M, Singh MK, Tyagi V. Ecologically sustainable integrated weed management in dry and irrigated direct-seeded rice. Advances in Plants & Agriculture Research. 2018; 8(4): 319-331. DOI: 10.15406/apar.2018.08.00333.
48. DWSR. Vision 2030. Directorate of Weed Science Research, Maharajpur, Adhartal Jabalpur, Published by Director, DWSR, Jabalpur. 2011; pp. 1-28.
49. Kewat ML. Improved weed management in Rabi crops. National Training on Advances in Weed Management. 2014; pp. 22-25.
50. Singh Mahender, Tomar IS, Singh VK, Garg SK. Effect of integrated weed management on yield of soybean (Glycine max. L.). Bhartiya Krishi Anusandhan Patrika. 2015; 30(4): 204-205.
51. Green JM, Owen MD. Herbicide-resistant crops: utilities and limitations for herbicide-resistant weed management. Journal of Agricultural and Food Chemistry. 2011; 59(11): 5819–5829. https://doi.org/10.1021/jf101286h.
52. Bineet M, Anda G, Mohamed TA. Herbicide mixture in agriculture: a review. In: Proceeding of Biennial Conference, Indian Society of Weed Science, held at Bangalore. 2001; pp. 236.

53. Lamichhane Jay Ram, Yann Devos, Hugh J, Beckie Micheal DK, Owen Pascal, Tillie Antoine, Messéanand Per Kudsk. Integrated weed management systems with herbicide-tolerant crops in the European Union: lessons learnt from home and abroad. Critical Reviews in Biotechnology. 2017; 37(4): 459-475. DOI: 10.1080/07388551.2016.1180588.
54. Vargas RN, Wright SD. UC IPM Pest Management Guidelines: Cotton. State wide IPM Program, Division of Agriculture and Natural Resources, University of California, Publication 3444, Oakland. 2005.
55. Gill GS, Kumar VK. Weed index–A new method for reporting weed control trails. Indian Journal of Agronomy. 1969; 14: 96-98.
56. Jackson ML. Soil chemical analysis, New Delhi: Asian Publication House. 1958; pp. 173–195.
57. Chapman HD, Pratt PF. Methods of analysis for soils, plants and waters. Division of Agric. Sci., Berkeley, Univ. California, USA. 1961; pp. 150–152.
58. Singh D, Chhonkar PK, Dwivedi BS. Manual on soil, plant and water analysis. Westville publishing house, New Delhi, India. 2005; pp. 1–199. https://www.cabdirect.org/cabdirect/abstract/20073118818.
59. Jackson ML. Soil chemical analysis, New Delhi: Prentice Hall. 1973; pp. 498.
60. Kalhapure AH, Shete BT, Pendharkar AB, Dhage AB, Gaikwad DD. Integrated weed management in soybean. Journal of Agriculture Research and Technology. 2011; 36(2): 217-219.
61. Mishra JS, Singh VP. Weed dynamics and productivity of soybean (Glycine max) - based cropping systems as influenced by tillage and weed management. Indian Jounal of Agronomy. 2009; 54(1): 29-35.
62. Malik RS, Yadav A, Malik RK. Integrated weed management in soybean (Glycine max L.). Indian Journal of Weed Science. 2006; 38(1 and 2): 65-68.
63. Chandel AS, Saxena SC. Effect of some new post emergence herbicides on weed parameters and seed yield of soybean. Indian Journal of Agronomy. 2001; 46(2): 332-338.
64. Kumar S, Rana MC, Rana SS, Sharma A. Effect of propaquizafop alone and in mixture with other herbicides on weed dry weight and growth and yield of soybean. Journal of Crop and Weed. 2018; 14(2): 149-153.
65. Prachand S, Kalhapura A, Kubde KJ. Weed management in soybean with pre and post emergence herbicides. Indian Journal of Weed Science. 2015; 47(2): 163-165.

66. Singh VP, Barman KK, Singh Raghwendra, Singh PK, Sharma AR. Weed management in conservation agriculture system. ICAR - Directorate of Weed Research, Jabalpur, India. 2015; pp. 1-60.

67. Muoni T, Rusinamhodzi L, Thierfelder C. Weed control in conservation agriculture systems of Zimbabwe: Identifying economical best strategies. Crop Protection. 2013; 53: 23-28.

68. Patel BD, Khedkar HP, Patel RB, Sheta BT. Bio efficacy of some selective post emergence herbicide in kharif soybean. Research on Crops. 2011; 12(2): 405-408.

69. Sharma PB. Quizalofop ethyl: An efficient herbicide, against grassy weeds of soybean. Pestology. 2000; 24(4): 60-62.

70. Raskar BS, Bhoi PG. Bio-efficacy and phytotoxicity of pursuit plus herbicides against weeds in soybean (Glycine max L.). Indian Journal of Weed Science. 2002; 34(1 and 2): 50-52.

71. Shrestha A, Lanini T, Wright S, Vargas R, Mitchell J. Conservation tillage and weed management. ANR publication 8200. 2006. http://www.anrcatalog.ucdavis.edu.

72. Wang Xiaobin, Cai Dianxiong, Hoogmoed WB, Oenema O, Perdoc UD. Scenario analysis of tillage, residue and fertilization management effects on soil organic carbon dynamics. Pedosphere. 2005; 15(4): 478-483.

73. Burton J, Chen C, Xu Z, Ghadiri H. Gross nitrogen transformationsin adjacent native and plantation forests of subtropical Australia. Soil Biology and Biochemistry. 2007; 39: 426–433. https://doi.org/10.1016/j.soilbio.2006.08.011.

74. Yadav D, Shivay YS, Singh YV, Sharma VK, Bhatia A. Enhancing nutrient translocation, yields and water productivity of wheat under rice–wheat cropping system through zinc nutrition and residual effect of green manuring. Journal of Plant Nutrition. 2020; 43: 2845–2856. https://doi.org/10.1080/01904167.2020.1798997.

75. Ojeda G, Mattana S, Àvila A, Alcañiz JM, Volkmann M, Bachmann J. Are soil-water functions affected by biochar application? Geoderma. 2015; 249: 1–11. https://doi.org/10.1016/j.geoderma.2015.02.014.

76. Gong X, Liu Y, Li Q, Wei X, Guo X, Niu D, Zhang L. Subtropical degraded red soil restoration: Is soil organic carbon build-up limited by nutrients supply. Forest Ecology and Management. 2013; 300: 77–87. https://doi.org/10.1016/j.foreco.2012.12.002.

77. Meena BP, Biswas AK, Singh M, Chaudhary RS, Singh AB, Das H, Patra AK. Long-term sustaining crop productivity and soil health in maize–chickpea system through integrated nutrient management

practices in Vertisols of central India. Field Crops Research. 2019; 232: 62-76. https://doi.org/10.1016/j.fcr.2018.12.012.

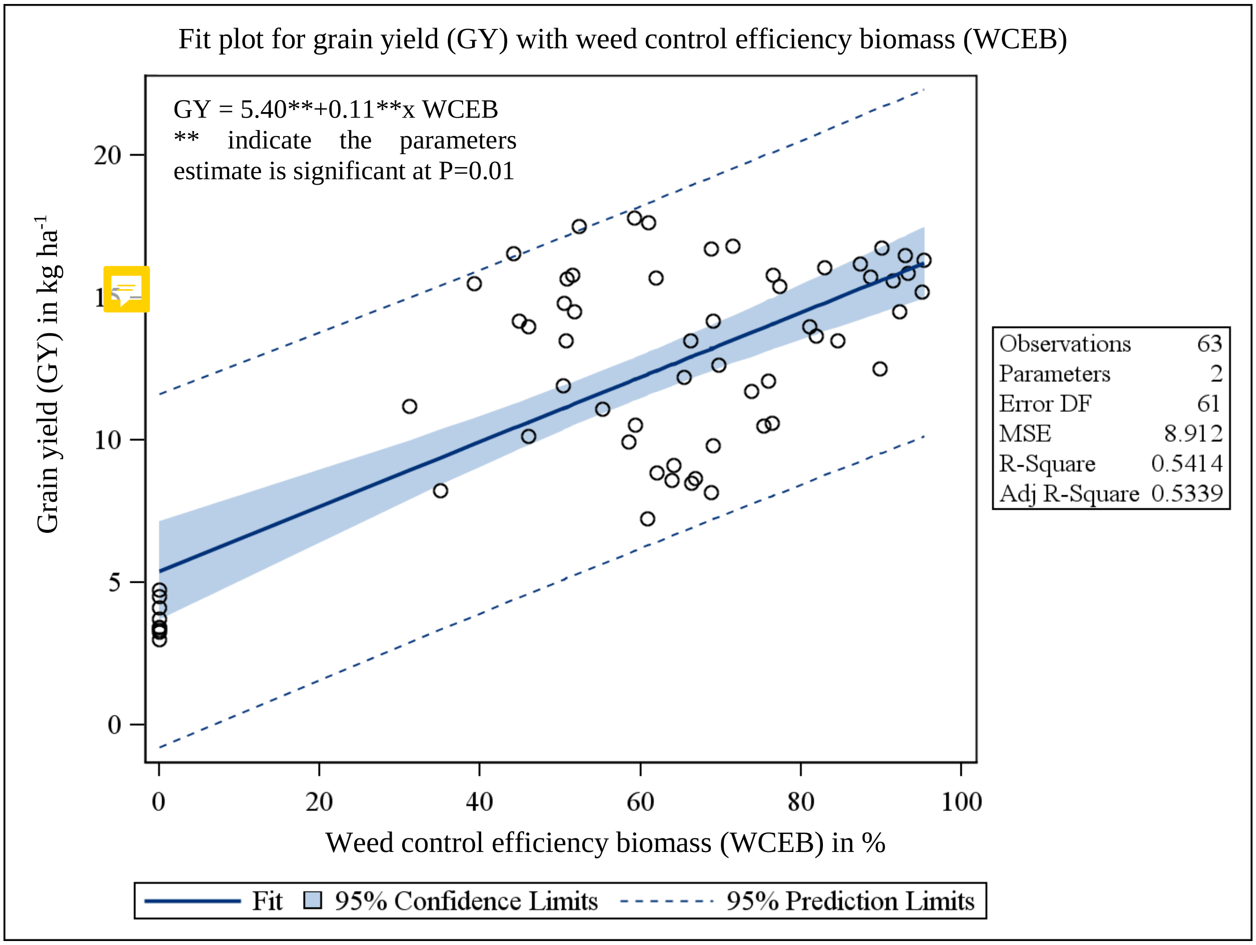
Fit plot for grain yield (GY) with weed control efficiency biomass (WCEB)
GY = 5.40**+0.11**x WCEB
** indicate the parameters estimate is significant at P=0.01
Grain yield (GY) in kg ha-1
20
10
5
0
0
20
40
60
80
100
Weed control efficiency biomass (WCEB) in %
Observations 63
Parameters 2
Error DF 61
MSE 8.912
R-Square 0.5414
Adj R-Square 0.5339
Fit
95% Confidence Limits
95% Prediction Limits

Figure 1.

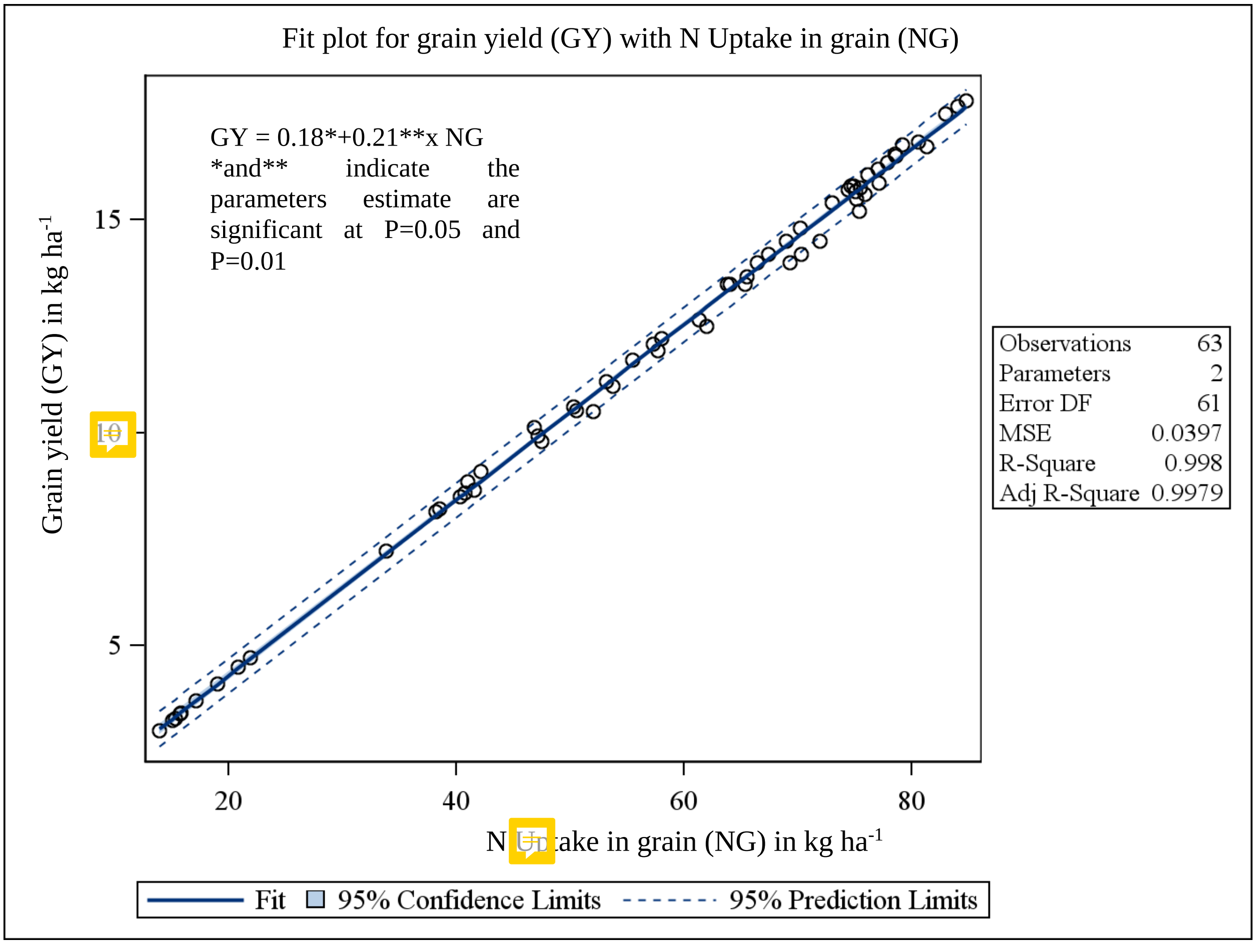
Fit plot for grain yield (GY) with N Uptake in grain (NG)
GY = 0.18*+0.21**x NG
*and** indicate the parameters estimate are significant at P=0.05 and P=0.01
Grain yield (GY) in kg ha-1
15
10
5
20
40
60
80
N Uptake in grain (NG) in kg ha-1
Observations 63
Parameters 2
Error DF 61
MSE 0.0397
R-Square 0.998
Adj R-Square 0.9979
Fit
95% Confidence Limits
95% Prediction Limits

Figure 2.

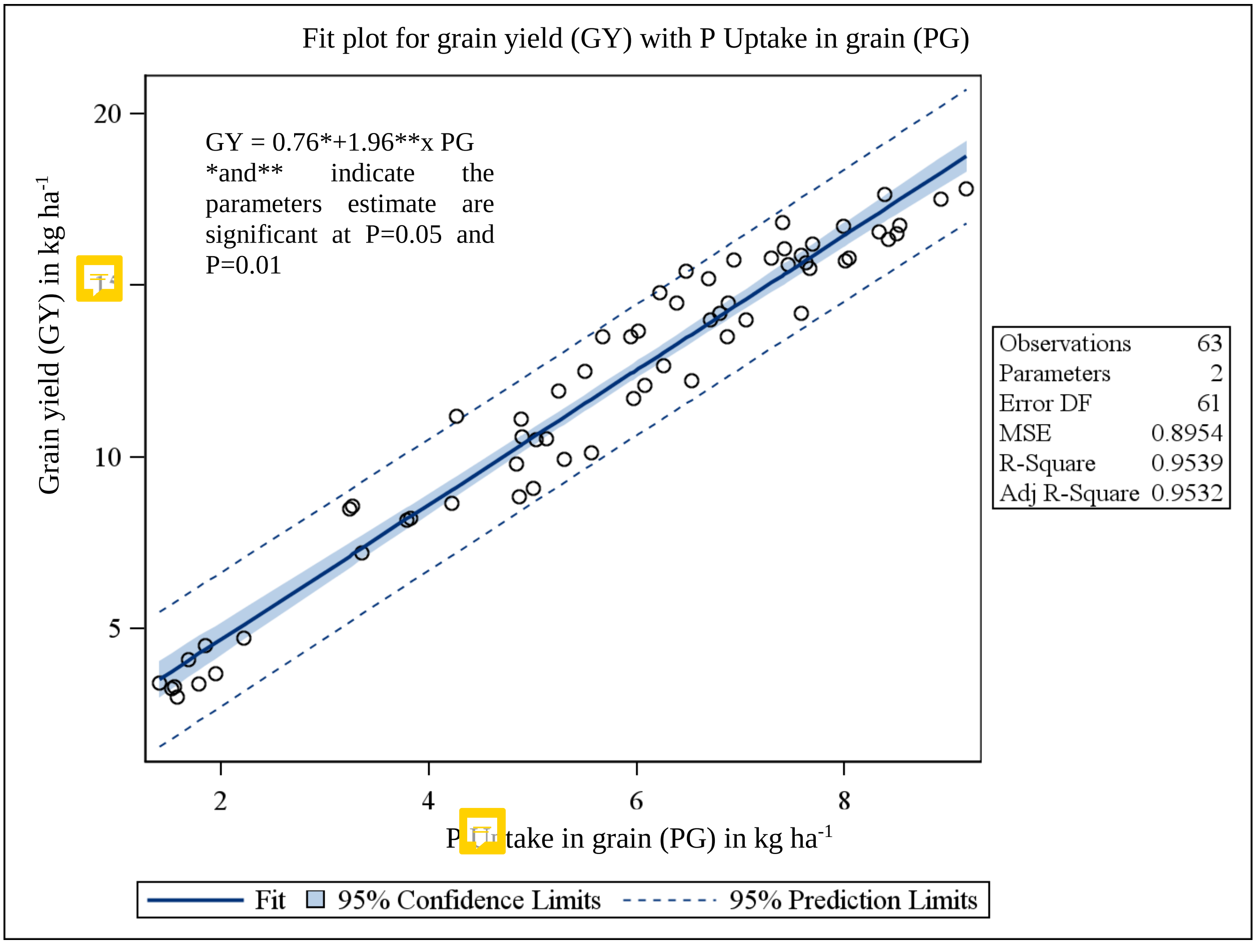
Fit plot for grain yield (GY) with P Uptake in grain (PG)
GY = 0.76*+1.96**x PG
*and** indicate the parameters estimate are significant at P=0.05 and P=0.01
Grain yield (GY) in kg ha-1
20
10
5
2
4
6
8
take in grain (PG) in kg ha-1
Observations 63
Parameters 2
Error DF 61
MSE 0.8954
R-Square 0.9539
Adj R-Square 0.9532
Fit
95% Confidence Limits
95% Prediction Limits

Figure 3.

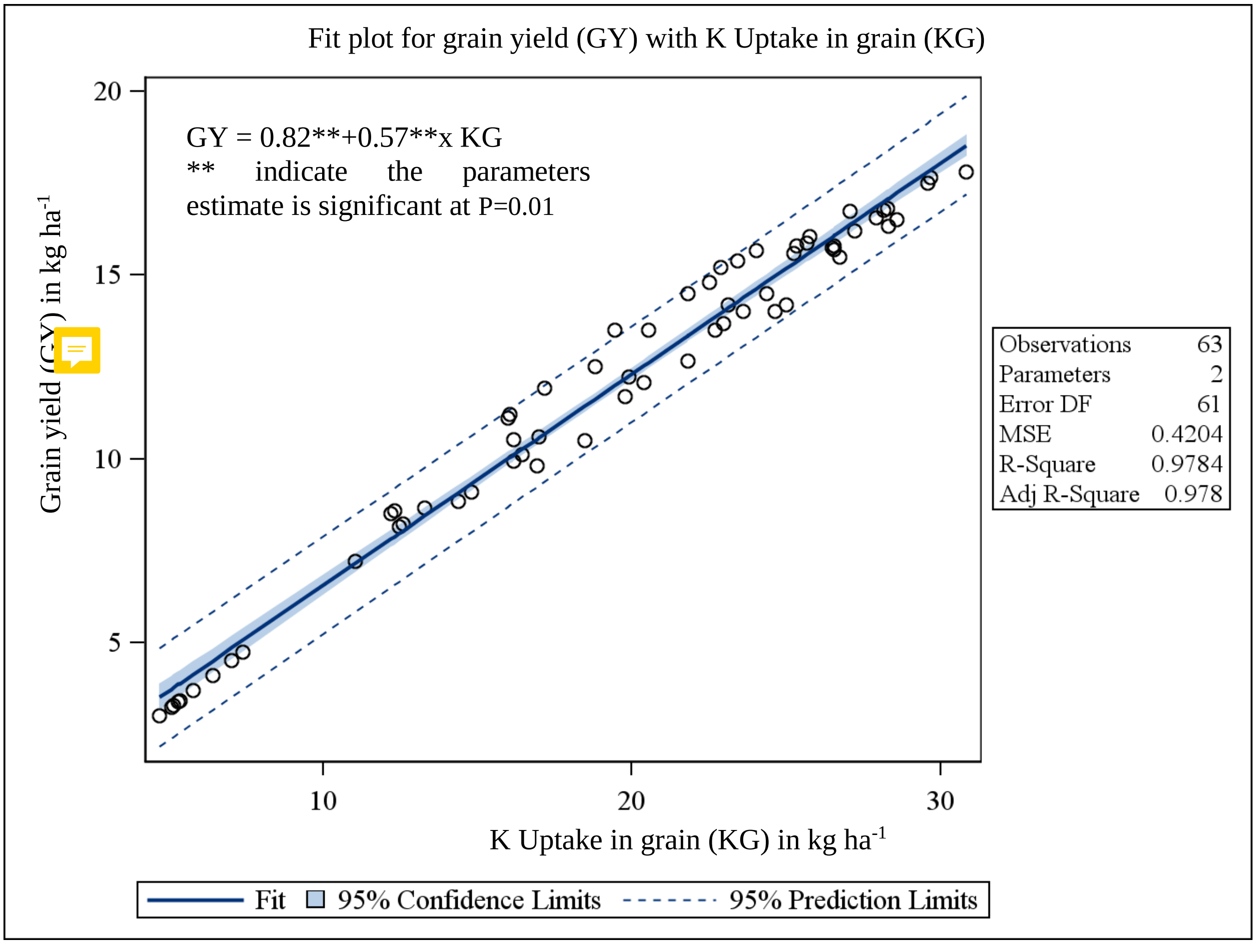

Figure. 4

Figure captions

Fig 1. Relationship between grain yield (GY) and weed control efficiency biomass (WCEB) under herbicidal weed management practices in soybean.

Fig 2. Relationship between grain yield (GY) and N Uptake in grain (NG) under herbicidal weed management practices in soybean.

Fig 3. Relationship between grain yield (GY) and P Uptake in grain (PG) under herbicidal weed management practices in soybean.

Fig 4. Relationship between grain yield (GY) with K Uptake in grain (KG) under herbicidal weed management practices in soybean.
