# Supplementary material for: Impact of sequential herbicides application on crop productivity, weed and nutrient dynamics in soybean under conservation agriculture in Vertisols of Central India
Source: PLoS One. 2023 Jan 20;18(1):e0279434. doi: 10.1371/journal.pone.0279434 (PMC9857987; doi:10.1371/journal.pone.0279434)
Supplement: Attachment — Submitted filename: Comments.docx [file pone.0279434.s001.docx]

**Comments for Editor**

I have gone through the whole manuscript (MS) very thoroughly and found that author written MS as per the international standard to present the work before global research platform. I can see that author explained the study to evaluate the different herbicide combinations under conservation agriculture in soybean crop particularly in rainfed ecosystem. Since, adoption of conservation agriculture (CA) is very leisurely due to high weed infestations throughout the globe. Hence, author selected an important issue of weed management in CA, which is globally spread and its management needs basic and applied research to increase area of the CA. The research hypothesis addressed applied aspect of research. Based on experimental result, it is a scientifically sound and may be accepted after minor revision. However, some of the minor corrections/suggestions are given below.

**Comments for Authors**

- Abstract written very well however some minor correction need to be incorporated like how much per-chant age increase in crop productivity under the best system.
- Introduction: Introduction is very well written indicating about background and hypothesis of study about weed management under conservation agriculture. However, it can be condensed as it is very largely elaborated.
- Materials and methods: This section described methods of all parameters and can be included some spelling mistakes with some minor correction and modifications.
- Whether the crop variety (JS-335, soybean) used same in all years? and author performed the field study under rainfed ecosystem particularly under conservation agriculture, whether the used crop variety was screen out for the conservation agriculture?
- Soybean crop is very susceptible to insect and pest attack, what type plant protection measures were used to check the pest problem.
- Nitrogen, phosphorus and potassium can be written as N, P and K as it is well understood, and no need to write again and again.
- Recommended rate of fertilizer (30 kg N+ 60 kg P_2_O_5_ +40 kg K_2_O ha^-1^) has been drilled uniformly at the time of sowing with ‘happy seeder’ as basal application, whether, it was drilled or broadcast or sprayed, check it again ?.
- Results: Tables and figures were explained very well in result section, some minor modification is needed to be corrected.
- “Please check the Latin names of the weeds?.
- Discussion: I appreciate the discussion part, the way author discussed the results with other findings is quite good. Only I have a small query that author about the 30% residue level in all the plots irrespective of herbicidal treatments, whether it was ‘anchored’ residue or residue ‘incorporation’, please mentioned it clearly.

Conclusion is written well although there is scope to improve it in briefly as per salient achievement of the study.

We concluded….. replace with it can be concluded.

- References: All the cited references in text are in reference list and also as per the journal’s format, but please check it carefully again.
- English grammar and punctuation is very good throughout the manuscript and the study accompanying results have merit but minor revisions are needed to make this manuscript worthy of publication.
