# Supplementary material for: Impact of sequential herbicides application on crop productivity, weed and nutrient dynamics in soybean under conservation agriculture in Vertisols of Central India
Source: PLoS One. 2023 Jan 20;18(1):e0279434. doi: 10.1371/journal.pone.0279434 (PMC9857987; doi:10.1371/journal.pone.0279434)
Supplement: Attachment — Submitted filename: Review comments Manuscript.docx [file pone.0279434.s003.docx]

**Manuscript No.** - PONE-D-22-19003

**Title:** Impact of sequential herbicides………in Vertisols of Central India

**Comments:** Conservation Agriculture (CA) is a relatively new field in agricultural research but it is the need of the hour for sustainable production and enhancing the income of resource poor farming communities across the globe. The present study deals with the sequential application of all suitable and available herbicides for successful raising of field crop in Kharif season. Managing the weeds in the field is a major challenge and this becomes further daunting task in rainy season and with no-till practices. Therefore, the present study is very much required and novel one.

Decision: This manuscript can be published in PLOS ONE Journal with minor corrections as suggested below:

- Scientific names of few weed spp. need to be rechecked.
- There are few typological errors
- Few new and relevant references can be added, if possible
- There are few errors occurred during conversion to PDF.
