# Supplementary material for: Impact of sequential herbicides application on crop productivity, weed and nutrient dynamics in soybean under conservation agriculture in Vertisols of Central India
Source: PLoS One. 2023 Jan 20;18(1):e0279434. doi: 10.1371/journal.pone.0279434 (PMC9857987; doi:10.1371/journal.pone.0279434)
Supplement: Attachment — Submitted filename: Response to reviewers.docx [file pone.0279434.s004.docx]

**Journal Name:** PONE-D-22-19003

**Manuscript title**: Impact of sequential herbicides application on crop productivity, weed and nutrient dynamics in soybean under conservation agriculture in Vertisols of Central India

**Dear Editor**,

Thank you for your useful comments and suggestions on the structure of our manuscript. We are very thankful to the reviewer for thoroughly reviewing our manuscript. These comments and suggestions were helpful in improving our manuscript and we have addressed them in the revised version and highlighted with red color.

Here we are also providing detailed responses to the reviewers’ comments.

|  | **Comments** | **Response** |
| --- | --- | --- |
|  | **Reviewer 1** |  |
| 1. | Abstract written very well however some minor correction need to be incorporated like how much per-chant age increase in crop productivity under the best system. | Agreed and complied,  Thanks for appreciating of our efforts.  Incorporated in the revised manuscript as per suggestions. |
| 2. | Introduction: Introduction is very well written indicating about background and hypothesis of study about weed management under conservation agriculture. However, it can be condensed as it is very largely elaborated. | Thank you very much for appreciating of our efforts. |
| 3. | Materials and methods: This section described methods of all parameters and can be included some spelling mistakes with some minor correction and modifications. | Agreed and corrected all the mistakes and incorporated in the revised manuscript as per suggestions. |
| 4 | Whether the crop variety (JS-335, soybean) used same in all years? and author performed the field study under rainfed ecosystem particularly under conservation agriculture, whether the used crop variety was screen out for the conservation agriculture? | Yes, same varieties were used for conducting all season experiment and variety and included in the material and method section of the manuscript. |
| 5 | Soybean crop is very susceptible to insect and pest attack, what type plant protection measures were used to check the pest problem. | Imidacloprid and thiamethoxam were sprayed to control aphids, thrips and whiteflies while Emamectin benzoate was used to control lepidopteran pests. |
| 6 | Nitrogen, phosphorus and potassium can be written as N, P and K as it is well understood, and no need to write again and again. | Thank you very much for your valuable suggestions on our manuscript  Agreed and complied. |
| 7 | Recommended rate of fertilizer (30 kg N+ 60 kg P_2_O_5_ +40 kg K_2_O ha^-1^) has been drilled uniformly at the time of sowing with ‘happy seeder’ as basal application, whether, it was drilled or broadcast or sprayed, check it again ?. | Agreed, complied and corrected as per suggestions. |
| 8 | Results: Tables and figures were explained very well in result section, some minor modification is needed to be corrected. | Agreed and complied,  Thanks for appreciating of our efforts Incorporated as per suggestions. |
| 9 | Please check the Latin names of the weeds?. | Thank you much for pointing out. All the Latin names of the weeds are checked and corrected as per suggestions. |
| 10 | Discussion: I appreciate the discussion part, the way author discussed the results with other findings is quite good. Only I have a small query that author about the 30% residue level in all the plots irrespective of herbicidal treatments, whether it was ‘anchored’ residue or residue ‘incorporation’, please mentioned it clearly. | Thank you very much for appreciating our efforts. |
| 11 | Conclusion is written well although there is scope to improve it in briefly as per salient achievement of the study. | Thank you very much for your positive comments on our manuscript.  Agreed, the conclusion revised as per suggestions. |
| 12. | We concluded….. replace with it can be concluded. | Agreed, replaced as per suggestions. |
| 13 | References: All the cited references in text are in reference list and also as per the journal’s format, but please check it carefully again. | Checked all the references and cited as per Journal format. |
| 14 | English grammar and punctuation is very good throughout the manuscript and the study accompanying results have merit but minor revisions are needed to make this manuscript worthy of publication. | Agreed, the grammar and punctuation again also checked and corrected wherever required. Thanks for appreciating. |
| **Reviewer #2:** | | |
| 1 | Authors have attempted a good piece of work for continuous three years to manage the weeds in soybean under CA.  However, the manuscript need a major revision, | Agreed and complied,  Thanks for appreciating of our efforts. The per-chant increase in crop productivity under the best is incorporated as per suggestions. |
| 2 | The conclusion part needs to be rewritten given the emphasis on the findings. | Thank you very much for your positive comments on our manuscript. Based on your comments, we have discussed and revised the contents of our conclusion. The purpose is to make our conclusions emphasize the scientific value of our research and highlight the innovation and novelty of our research. |
| 3 | In the figures, y-axis (yield parameters) values need to be checked and changed accordingly. | Agreed and checked as per suggestions. |
| 4 | The rest of the comments have been appended to the body of the text. | All the comments given in the manuscript are addressed and corrected in revised manuscript |
| **Reviewer #3:** | | |
| 1 | Researchers have conducted study on to  evaluate the expediency of different herbicides and their sequential applications under CA. The manuscript is well written, explained and discussed well, however, I am having few queries and should be incorporated it in manuscript before final decision. | Thank you very much for appreciating of our efforts. And all the queries are checked and corrected as per given suggestions. |
| 2 | The study was conducted for 3 years and data related to weed density, weed dry matter and grain yield was provided as a mean or average of three years, isn't it? You have rightly mentioned in the introduction section that the effect of CA is visible in longer run, here; I want to see the year wise data of weed density, weed dry matter, species wise difference and grain yield for year wise. I believe it will improve the quality of manuscript and easy to understandable for international readers.  Kindly incorporate it? | Thank you very much for your valuable comments on our manuscript. We carefully and detailed studied of the given comments. However, at this junction it may not be possible to incorporate yearly data as it will change the basic framework of the manuscript. |
| 3 | Methodology of agronomic efficiency and PUE is missing in respective section, incorporate it? | Thank you very much for your valuable comments on our manuscript and Incorporated the missing methodology of agronomic efficiency and PUE in the revised manuscript. |
|  | Control plot- you mean herbicide was not applied in control treatment or nutrients were not applied, elaborate it? | Agreed, herbicides not applied in control plots however, RDF was applied uniformly. |
| **R**eviewer #4: | |  |
|  | Scientific names of few weed spp. need to be rechecked. | All the scientific names are checked and corrected in the manuscript. |
|  | There are few typological errors• | All the grammatical and typological errors are corrected in the revised manuscript. |
|  | Few new and relevant references can be added, if possible | Agreed, included in the revised manuscript. |
|  | There are few errors occurred during conversion to PDF. | Thank you very much for your valuable suggestions on our manuscript. All the errors are checked and corrected as per given suggestions. |

The revised and corrected manuscript has been submitted to your journal. We look forward to your positive response.
