# Supplementary material for: Impact of sequential herbicides application on crop productivity, weed and nutrient dynamics in soybean under conservation agriculture in Vertisols of Central India
Source: PLoS One. 2023 Jan 20;18(1):e0279434. doi: 10.1371/journal.pone.0279434 (PMC9857987; doi:10.1371/journal.pone.0279434)
Supplement: Attachment — Submitted filename: response to reviewers.docx [file pone.0279434.s005.docx]

**Response to editors’ comments**

Thank you for your useful comments and suggestions on the structure of our manuscript. We are very thankful to the reviewer for thoroughly reviewing our manuscript. These comments and suggestions were helpful in improving our manuscript and we have addressed them in the revised version and highlighted with red color. All the editorial comments have been incorporated throughout the manuscript text as pointed out. Checked all grammatically error, spelling of heading and subheading key words of the manuscript. Retracted references are deleted and revised in both the references list and the manuscript text.

Here we are also providing detailed responses to the reviewers’ comments.

**Response to reviewers’ comments**

**Comment 1:** Manuscript has been significantly improved by the authors and it looks in good shape to accept for publication.

**Response**: Thanks for your endurance and appreciating of our efforts.

**Comment 2:** The issue addressed in manuscript is very much pertaining to soybean cultivation in vertisol of Central India and similar agro-ecosystem.

**Response**: Thanks for appreciating of our efforts.

**Comment: 3** In recent past, many of the weed species has shown tolerant to ruling herbicides. Thus, information generated with this study will be useful to soybean growers and similar group crops (greengram and blackgram).

**Response**: Thanks for appreciating our hypothesis

**Comment** 4: However, in my previous review I have suggested to modify for figures Y axix, but I did not see it in present attached documents. This might be due to non-submission or not attached.

**Response**: Thanks for valuable advice and comments. The figures have been changed and the Y axis values have been corrected (yield data and unit, kg/ha) in the revised version of the figures.

**Comment 5:** Abbreviations and terminologies should be used appropriately throughout the manuscript.

**Response**: Indeed, abbreviations and terminologies are checked thoroughly and corrected as needed.

**Comment 6**: Spelling of scientific name of the weeds need to be re-checked.

**Response**: I thoroughly checked the entire manuscript for grammatical and spelling errors.

**Comment 7**: The weed density values need to be rounded off to nearest values, no need to present two digits after decimal.

**Response**: Thanks for valuable suggestions and the weed density values in table 2 is rounded as per given advice.

**Comment** 8: Weed index is yield dependent parameters so these may be presented beside yield. The suggested points may be looked critically.

**Response**: Agreed, the tables of weed index, weed biomass, and weed control efficiency (WCE) have been replaced with growth and yield parameters as per suggestion.

**Comment 9**: Rest of the comments are appended in the body itself for considerations.

**Response**: All comments have been addressed as appropriate.

**Comment 10**: In conclusions, supported with data might have further improved the standard of the manuscript.

**Response**: Conclusion is improved as per suggestions.
